# Avelumab + axitinib treatment in older patients with advanced renal cell carcinoma in Japan: Subgroup analyses of post‐marketing surveillance data by age

**DOI:** 10.1002/cam4.70186

**Published:** 2025-01-21

**Authors:** Mototsugu Oya, Taito Ito, Masashi Sato, Makiko Morita, Masahiro Kajita, Norio Nonomura

**Affiliations:** ^1^ Department of Urology Keio University School of Medicine Tokyo Japan; ^2^ Medical Department Merck Biopharma Co., Ltd., Tokyo, Japan, an Affiliate of Merck KGaA Darmstadt Germany; ^3^ Research and Development Merck Biopharma Co., Ltd., Tokyo, Japan, an Affiliate of Merck KGaA Darmstadt Germany; ^4^ Global Patient Safety Japan Merck Biopharma Co., Ltd., Tokyo, Japan, an Affiliate of Merck KGaA Darmstadt Germany; ^5^ Department of Urology Osaka University Graduate School of Medicine Osaka Japan

**Keywords:** checkpoint control, clinical cancer research, renal cancer, target therapy, tyrosine kinase inhibitors

## Abstract

**Introduction:**

Avelumab + axitinib was approved in Japan in December 2019 for the treatment of curatively unresectable or metastatic renal cell carcinoma (RCC) based on results from the JAVELIN Renal 101 trial.

**Materials and Methods:**

To evaluate the safety and effectiveness of avelumab + axitinib in older patients in general clinical practice in Japan, an ad hoc analysis of data from post‐marketing surveillance (PMS) by age group was conducted.

**Results:**

The analysis population included 328 patients who had received ≥1 dose of avelumab and were enrolled between December 2019 and May 2021. In total, 100 patients (30.5%) were aged ≤64 years, 130 (39.6%) were aged 65–74 years, and 98 (29.9%) were aged ≥75 years. Within these age groups, adverse drug reactions (ADRs) of safety specifications of any grade occurred in 46 (46.0%), 71 (54.6%), and 56 (57.1%), and of grade ≥3 in 13 (13.0%), 23 (17.7%), and 20 (20.4%), respectively. The most common ADRs of safety specifications across all age groups were thyroid dysfunction, infusion reactions, and hepatic function disorders. Median overall survival (OS) was not reached in any age group; 12‐month OS rates in patients aged ≤64, 65–74, or ≥75 years were 83.8%, 86.2%, and 80.0%, and objective response rates were 31.0%, 43.8%, and 30.6%, respectively.

**Discussion:**

Analyses of PMS data show the safety and effectiveness of avelumab + axitinib across all age groups of patients with RCC in general clinical practice in Japan. The favorable benefit–risk profile was generally consistent with that observed in previous clinical trials.

## Graphical abstract

We report analyses from post‐marketing surveillance of avelumab + axitinib treatment in patients with advanced renal cell carcinoma in Japan in age subgroups: ≤64 years (*n* = 100), 65–74 years (*n* = 130), and ≥75 years (*n* = 98). Overall, safety and effectiveness were similar across age subgroups, suggesting that age alone is not a relevant consideration for avelumab + axitinib treatment. The favorable benefit–risk profile was generally consistent with that observed in previous clinical studies, further supporting the use of avelumab + axitinib treatment in this disease setting.

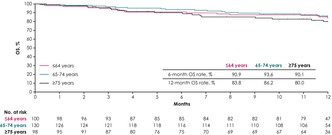

## 1 INTRODUCTION

In Japan in 2022, the kidney and other urinary organs were the ninth most frequent cancer site, with approximately ≈31,000 new cases and ≈10,000 deaths due to these cancers.^1^ Renal cell carcinoma (RCC) represents ≈80% of kidney cancers.^2^ The first‐line (1L) treatment landscape for advanced RCC (aRCC) has expanded in recent years with the introduction of immune checkpoint inhibitor (ICI)–based combination regimens, including ICI + tyrosine kinase inhibitors (TKI) combinations.^3^, ^4^, ^5^, ^6^, ^7^

Avelumab is an ICI that inhibits PD‐L1 and axitinib is a multitargeted TKI that inhibits vascular endothelial growth factor receptor.^8^, ^9^, ^10^, ^11^ Use of avelumab + axitinib as 1L treatment for aRCC was first evaluated in the JAVELIN Renal 100 phase 1b study, which provided evidence of antitumor activity and safety that was maintained with long‐term follow‐up (5 years).^12^, ^13^ The approval of avelumab + axitinib in various countries worldwide followed the results of the JAVELIN Renal 101 phase 3 trial (NCT02684006).^4^, ^8^, ^9^, ^10^, ^11^, ^14^, ^15^ After extended follow‐up (≥28 months in all patients), median progression‐free survival with avelumab + axitinib versus sunitinib in the overall study population was 13.9 versus 8.5 months (hazard ratio [HR], 0.67 [95% confidence interval {CI}, 0.568–0.785]; *p* < 0.0001), and median overall survival (OS) was not reached versus 37.8 months (HR, 0.79 [95% CI, 0.643–0.969]; *p* = 0.0116), respectively; the analysis of OS remained immature and follow‐up was ongoing until the final analysis.^15^

In subgroup analyses from JAVELIN Renal 101 in patients enrolled in Japan (*n* = 67) who were treated with avelumab + axitinib or sunitinib, median progression‐free survival was 16.6 versus 11.2 months (HR, 0.66 [95% CI, 0.296–1.464]), median OS was not reached in both arms (HR, 0.53 [95% CI, 0.042–6.693]), and objective response rates (ORRs) were 60.6% versus 17.6%, respectively.^16^ Based on these findings, avelumab + axitinib was approved in Japan for the treatment of patients with curatively unresectable or metastatic RCC in December 2019.^17^ This approval is supported by current Japanese Urological Association clinical practice guidelines for RCC (updated in 2022), which recommend avelumab + axitinib for 1L treatment of patients with clear cell RCC.^18^

Because of the limited number of Japanese patients who were enrolled in JAVELIN Renal 101, post‐marketing surveillance (PMS) was required to further evaluate the safety and effectiveness of avelumab + axitinib treatment in patients with RCC in general clinical practice in Japan. Initial analyses showed the safety and effectiveness of avelumab + axitinib in the overall PMS population.^19^ Because Japan has a super‐aging population, including a higher proportion of individuals aged ≥65 years than most countries,^20^ real‐world data in older patients are highly relevant. In a subgroup analysis from the JAVELIN Renal 101 trial, avelumab + axitinib demonstrated favorable efficacy and consistent tolerability versus sunitinib in subgroups of patients aged <65 years, ≥65–74 years, and ≥75 years.^21^ The Ministry of Health, Labour and Welfare (MHLW) of Japan defines patients aged 65–74 years as “early‐stage elderly” and patients ≥75 years as “late‐stage elderly.” Here, we report an ad hoc analysis of PMS data from Japan in patients with aRCC by age group: <65 years, ≥65–74 years, and ≥75 years.

## 2 MATERIALS AND METHODS

### 2.1 Study design and patient population

Full details of the PMS design for avelumab + axitinib in RCC are reported in a separate manuscript.^19^ In brief, prospective, noncomparative, observational PMS was conducted at multiple centers throughout Japan to evaluate the safety and effectiveness of avelumab + axitinib treatment in patients with RCC. The PMS population included all patients with curatively unresectable or metastatic RCC who received ≥1 dose of avelumab and who were enrolled from December 20, 2019 (date of regulatory approval), to May 31, 2021. The observation period for all patients was ≤52 weeks. In the current report, subgroup analyses were performed in patients aged ≤64, 65–74, or ≥75 years. Investigators completed electronic or paper case report forms, and data were collected and analyzed by the sponsor. The PMS followed Japanese regulations for Good Post‐Marketing Study Practice.^22^ The protocol was reviewed by all participating institutions, and patients provided written informed consent based on the institutional requirements.

### 2.2 Endpoints

The primary objective of the PMS was to evaluate the safety of avelumab + axitinib. Data on adverse drug reactions (ADRs) of safety specifications deemed to have a causal relationship with avelumab were collected. ADRs of safety specifications included the following: interstitial lung disease, pancreatitis, hepatic function disorders, colitis/severe diarrhea, thyroid dysfunction, adrenal insufficiency, pituitary disorders, type 1 diabetes mellitus, myocarditis, nerve disorders (including Guillain–Barré syndrome), renal disorders, myositis/rhabdomyolysis, infusion reaction, encephalitis/meningitis, and myasthenia gravis.

The secondary objective was to evaluate the effectiveness of avelumab + axitinib. Effectiveness endpoints were best overall response and OS. Best overall response (i.e., complete response, partial response, stable disease, progressive disease, or not evaluable) was determined by investigators with reference to RECIST v1.1.^23^ ORR (best response of complete response or partial response) and disease control rate (DCR; best response of complete response, partial response, or stable disease) were calculated. OS was defined as the time from start of avelumab + axitinib treatment until death from any cause.

### 2.3 Statistical analyses

Definitions of safety and effectiveness analysis populations are reported in a separate manuscript.^19^ Safety data were aggregated by system organ class and preferred term and presented as overall frequency and percentages, in addition to worst grade. For best overall response, proportions of patients with complete response, partial response, stable disease, progressive disease or not evaluable were calculated. For ORR and DCR, proportions of patients were calculated with 95% CIs. OS was estimated using Kaplan–Meier methodology, including median, rates at time points, and 95% CIs. Patients who were alive or lost to follow‐up at the end of the observation period were censored at the last observation date.

## 3 RESULTS

### 3.1 Patients and treatment

At database lock (March 22, 2023), 328 patients were included in the safety and effectiveness analysis populations. Of these, 100 (30.5%) were aged ≤64 years, 130 (39.6%) were aged 65–74 years, and 98 (29.9%) were aged ≥75 years (Table 1). The subgroup of patients aged ≥75 years included a lower proportion of patients with ECOG PS of 0, a higher proportion with ECOG PS of 1, a lower proportion with a favorable International Metastatic Renal Cell Carcinoma Database Consortium (IMDC) risk classification and a lower proportion who had undergone prior surgery, compared with other age subgroups. In addition, the subgroup of patients aged ≤64 years included a lower proportion of patients with renal impairment.

**TABLE 1:** Baseline characteristics by age group.

Characteristic	≤64 years (*n* = 100)	65–74 years (*n* = 130)	≥75 years (*n* = 98)
Age, median (range), years	57 (12–64)	70 (65–74)	79 (75–89)
Sex, *n* (%)			
Male	83 (83.0)	89 (68.5)	65 (66.3)
Female	17 (17.0)	41 (31.5)	33 (33.7)
Weight, median (range), kg	65.3 (29.5–113.9)	61.4 (33.0–93.2)	54.7 (31.2–88.6)
ECOG PS, *n* (%)			
0	68 (68.0)	90 (69.2)	50 (51.0)
1	26 (26.0)	26 (20.0)	40 (40.8)
≥2	5 (5.0)	13 (10.0)	8 (8.2)
Unknown	1 (1.0)	1 (0.8)	0
Disease stage classification, *n* (%)			
III	8 (8.0)	11 (8.5)	10 (10.2)
IV	85 (85.0)	108 (83.1)	80 (81.6)
Other	1 (1.0)	2 (1.5)	1 (1.0)
Not reported	6 (6.0)	9 (6.9)	7 (7.1)
Metastatic disease, *n* (%)			
Yes	94 (94.0)	122 (93.8)	92 (93.9)
No	6 (6.0)	8 (6.2)	6 (6.1)
Pathological classification, *n* (%)			
Clear cell RCC	80 (80.0)	107 (82.3)	84 (85.7)
Non‐clear cell RCC	10 (10.0)	10 (7.7)	2 (2.0)
Other^a^	10 (10.0)	13 (10.0)	12 (12.2)
IMDC risk classification, *n* (%)			
Favorable	32 (32.0)	42 (32.3)	16 (16.3)
Intermediate (1 risk factor)	23 (23.0)	32 (24.6)	36 (36.7)
Intermediate (2 risk factors)	16 (16.0)	22 (16.9)	20 (20.4)
Poor	23 (23.0)	21 (16.2)	16 (16.3)
Other	6 (6.0)	13 (10.0)	10 (10.2)
Comorbidity, *n* (%)			
Renal impairment	17 (17.0)	40 (30.8)	31 (31.6)
Hepatic impairment	6 (6.0)	4 (3.1)	4 (4.1)
Interstitial lung disease	0	0	1 (1.0)
Autoimmune disease	2 (2.0)	2 (1.5)	2 (2.0)
Prior treatment history, *n* (%)			
Surgery	69 (69.0)	93 (71.5)	50 (51.0)
Radiation therapy	15 (15.0)	13 (10.0)	3 (3.1)

Abbreviations: ECOG PS, Eastern Cooperative Oncology Group performance status; IMDC, International Metastatic Renal Cell Carcinoma Database Consortium; RCC, renal cell carcinoma.

^a^ Information about RCC pathological classification was missing or unknown.

The median number of doses of avelumab received in the ≤64, 65–74, and ≥75 years subgroups was 14.5 (range, 1–26), 15.0 (range, 1–27), and 10.5 (range, 1–26), respectively (Table S1). The median duration of treatment, rates of dose reduction, dose escalation, and relative dose intensity for avelumab and axitinib are summarized in Table S1. At data cutoff, the number of patients who remained on treatment with avelumab and/or axitinib in the ≤64, 65–74, and ≥75 years subgroups was 33 (33.0%), 50 (38.5%), and 33 (33.7%), respectively. Rates of treatment discontinuation and reasons for discontinuation are summarized in Table S2.

### 3.2 Safety

**FIGURE 1:**
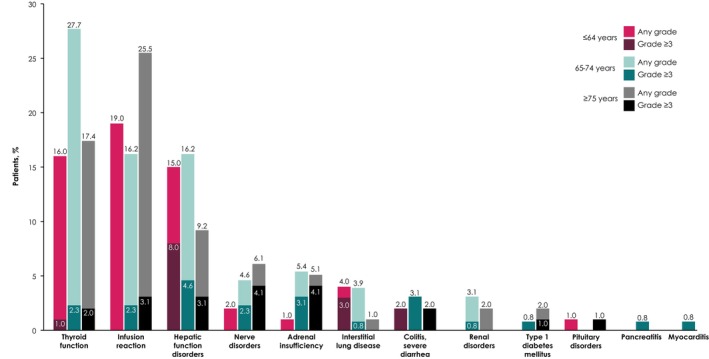
ADRs of safety specification of any grade by age group. ADR, adverse drug reaction.

**FIGURE 2:**
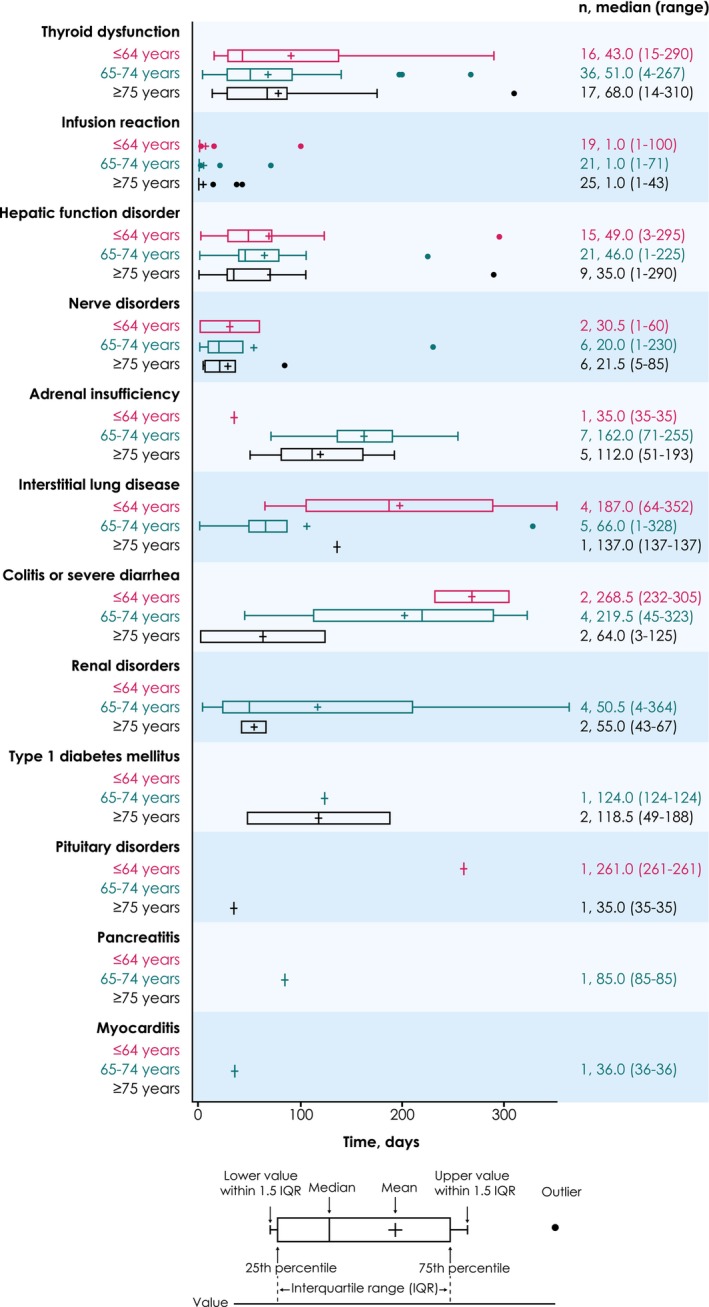
Time to onset of ADRs of safety specifications by age group. ADR, adverse drug reaction.

In the ≤64, 65–74, and ≥75 years subgroups, ADRs of safety specifications occurred in 46 (46.0%), 71 (54.6%), and 56 (57.1%), including grade ≥3 events in 13 (13.0%), 23 (17.7%), and 20 (20.4%), respectively (Table 2). ADRs led to death in one patient each in the 65–74 and ≥75 years subgroups (interstitial lung disease and Guillain–Barré syndrome, respectively). ADRs of safety specifications led to temporary discontinuation of avelumab in 9 patients (9.0%) aged ≤64 years, 24 patients (18.5%) aged 65–75 years, and 8 patients (8.2%) aged ≥75 years, and discontinuation of avelumab in 15 (15.0%), 19 (14.6%), and 24 (24.5%), respectively. Other reasons for treatment discontinuation are shown in Table S2. In the ≤64, 65–74, and ≥75 years subgroups, common ADRs of safety specifications (≥10% incidence) were thyroid dysfunction (16.0%, 27.7%, and 17.4%), infusion reaction (19.0%, 16.2%, and 25.5%), and hepatic dysfunction (15.0%, 16.2%, and 9.2%) (Figure 1). Time to onset of ADRs of safety specifications by age group is shown in Figure 2.

**TABLE 2:** Summary of ADRs of safety specifications by age group.

ADRs, *n* (%)	≤64 years (*n* = 100)	65–74 years (*n* = 130)	≥75 years (*n* = 98)
Any grade	46 (46.0)	71 (54.6)	56 (57.1)
Grade ≥3	13 (13.0)	23 (17.7)	20 (20.4)
Leading to death (grade 5)	0	1 (0.8)	1 (1.0)
Leading to interruption	9 (9.0)	24 (18.5)	8 (8.2)
Leading to dose reduction	2 (2.0)	1 (0.8)	1 (1.0)
Leading to discontinuation	15 (15.0)	19 (14.6)	24 (24.5)
Patients with high‐dose steroid treatment	6 (6.0)	10 (7.7)	8 (8.2)
Due to infusion reaction	2 (2.0)	4 (3.1)	6 (6.1)
Due to ADRs of safety specifications other than infusion reaction	5 (5.0)^a^	6 (4.6)	2 (2.0)

Abbreviation: ADR, adverse drug reaction.

^a^ One patient had both infusion reaction and an ADR of safety specifications other than infusion reaction.

Infusion reaction occurred at first dose in 17 patients (17.0%) aged ≤64 years, 19 patients (14.6%) aged 65–74 years, and 22 patients (22.4%) aged ≥75 years, including grade ≥3 events in 0, 2 (1.5%), and 3 (3.1%), respectively. Time of onset of first infusion reaction is shown in Table S3. Three patients aged 64–74 and three patients aged ≥75 years had >1 infusion reaction. In patients aged ≤64, 65–74, and ≥75 years, 99 (99.0%), 127 (97.7%), and 97 (99.0%) received premedication for infusion reactions at the first avelumab administration, respectively; among these patients, an infusion reaction occurred at first dose of avelumab in 17 (17.2%), 18 (14.2%), and 22 (22.7%).

In patients aged ≤64, 65–74, and ≥75 years, high‐dose steroids were administered following ADRs of safety specifications other than infusion reaction in 5 (5.0%), 6 (4.6%), and 2 (2.0%) and following infusion reaction in 2 (2.0%), 4 (3.1%), and 6 (6.1%), respectively (Table 2).

### 3.3 Effectiveness

**FIGURE 3:**
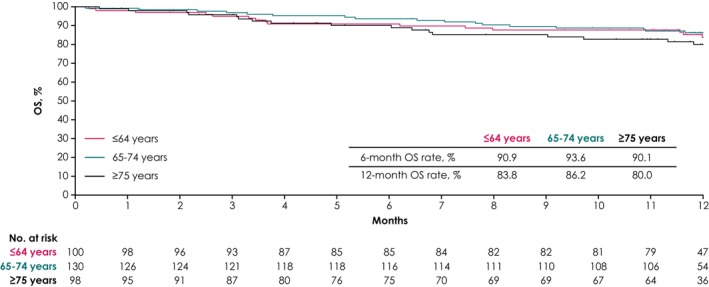
Kaplan–Meier analysis of OS by age group. OS, overall survival.

In the ≤64, 65–74, and ≥75 years subgroups, ORRs were 31.0% (95% CI, 22.1–41.0), 43.8% (95% CI, 35.2–52.8), and 30.6% (95% CI, 21.7–40.7), and DCRs were 73.0% (95% CI, 63.2–81.4), 79.2% (95% CI, 71.2–85.8), and 73.5% (95% CI, 63.6–81.9), respectively (Table 3). Median OS was not reached in any age subgroup. In patients aged ≤64, 65–74, and ≥75 years, 6‐month OS rates were 90.9%, 93.6%, and 90.1% and 12‐month OS rates were 83.8%, 86.2%, and 80.0%, respectively (Figure 3).

**TABLE 3:** Objective response by age group.

	≤64 years (*n* = 100)	65–74 years (*n* = 130)	≥75 years (*n* = 98)
Best overall response, *n* (%)			
Complete response	3 (3.0)	6 (4.6)	4 (4.1)
Partial response	28 (28.0)	51 (39.2)	26 (26.5)
Stable disease	42 (42.0)	46 (35.4)	42 (42.9)
Progressive disease	18 (18.0)	16 (12.3)	11 (11.2)
Not evaluable	0	0	2 (2.0)
Unknown	9 (9.0)	11 (8.5)	13 (13.3)
Objective response rate, *n* (%) [95% CI]	31 (31.0) [22.1–41.0]	57 (43.8) [35.2–52.8]	30 (30.6) [21.7–40.7]
Disease control rate, *n* (%), [95% CI]	73 (73.0) [63.2–81.4]	103 (79.2) [71.2–85.8]	72 (73.5) [63.6–81.9]

## 4 DISCUSSION

ICI + TKI combinations are important treatment options for aRCC.^3^, ^4^, ^6^, ^7^ Although avelumab + axitinib has been approved for the treatment of curatively unresectable or metastatic RCC in Japan since December 2019,^17^ real‐world evidence with this combination therapy in older patients is limited. This PMS provides a comprehensive dataset of patients with RCC who received avelumab + axitinib treatment in general clinical practice in Japan. Subgroup analyses reported here in patients aged <65, ≥65–74, and ≥75 years demonstrate the safety and effectiveness of avelumab + axitinib in patients with aRCC in general clinical practice irrespective of age.

The PMS population, compared with the avelumab + axitinib arm of the JAVELIN Renal 101 study,^21^ included a higher proportion of older patients, with 30.5% versus 61.3% aged ≤64 years, 39.6% versus 31.2% aged 65–74 years, and 29.9% versus 7.5% aged ≥75 years, respectively. In addition, the baseline characteristics of patients aged ≥75 years in the PMS population differed from those of other subgroups in ways that are expected for an older subgroup, such as worse performance status and a lower proportion with prior surgery, although most baseline characteristics were generally balanced across age groups.

The safety profile of avelumab + axitinib was comparable across age subgroups, including overall rates of ADRs of safety specifications of any grade (46.0%–57.1%) or grade ≥3 (13.0%–20.4%) and incidences of individual ADRs of safety specifications. ADRs appeared to be similarly manageable across subgroups, with high‐dose steroids required in 6.0%–8.2% of patients in each subgroup. Infusion reactions were more common during first administration in all age groups. ADRs of safety specifications led to temporary discontinuation or discontinuation of avelumab in a broadly similar proportion of patients in all age groups (8.2%–18.5% and 15.0%–24.5%, respectively). Overall, the safety results in the PMS population were comparable to clinical trial results, including the JAVELIN Renal 100 phase 1b trial and the JAVELIN Renal 101 phase 3 trial, which demonstrated the acceptable short‐ and long‐term safety profile of avelumab + axitinib in patients with aRCC.^4^, ^12^, ^13^, ^15^

ORRs in the ≤64, 65–74, and ≥75 years subgroups were 31.0%, 43.8%, and 30.6%, respectively, which are lower than the ORRs observed in age subgroups of the JAVELIN Renal 101 trial with avelumab + axitinib treatment (49.4%, 60.9%, and 42.4%, respectively).^21^ However, the PMS population was more heterogeneous than the JAVELIN Renal 101 population and included patients with ECOG PS 2, various comorbidities, and non‐clear cell pathological classification, who were not enrolled in JAVELIN Renal 101. Rates of PD as best response (11.2%–18.0%) and DCRs (73.0%–79.2%) in the PMS population were similar across age groups, indicating a similar degree of disease control in all age groups in general clinical practice. Median OS was not reached by data cutoff in any age subgroup, and 12‐month OS rates were generally consistent across age groups (80.0%–86.2%) and similar to the rate in the overall JAVELIN Renal 101 population treated with avelumab + axitinib (12‐month OS rate of 86%).^15^, ^24^

It has been hypothesized that immunotherapies might be less effective in older versus younger patients because of immunosenescence, a decline in immune activity in older patients that may contribute to cancer development because of a reduced capacity to combat carcinogenesis.^25^, ^26^ However, no reliable biomarkers for immunosenescence have been identified and this process may not be strictly linked to aging. ICI + TKI combination therapy utilizes complementary antitumor mechanisms, which may overcome immunosenescence in older patients. A meta‐analysis of randomized trials of 1L ICI‐based combinations for aRCC concluded that OS benefits were greater in younger patients.^27^ However, in an analysis reported by the IMDC, no efficacy differences were seen between older and younger patients with metastatic RCC receiving ICI‐based treatment in different treatment lines.^28^ Similarly, a comprehensive review of ICI treatment in older patients with aRCC concluded that available data did not indicate that efficacy was lower in older patients.^25^ In the PMS and JAVELIN Renal 101 populations, avelumab + axitinib showed no evidence of differences in safety and effectiveness across age groups, including patients aged ≥75 years.^21^ Overall, these studies suggest that potential immunosenescence in older patients is not a concern for avelumab + axitinib treatment in patients with aRCC.

The median relative dose intensity for avelumab was 100% in all age groups, whereas the median treatment duration was shorter in the ≥75 years subgroup versus other age subgroups (5.5 vs. 8.2–8.8 months). However, previous studies have shown that patients may continue to obtain treatment benefits after ICI discontinuation.^29^ The consistency in OS and DCR data between age subgroups supports the benefit of avelumab + axitinib treatment in older patients. Overall, these data suggest that clinical outcomes in younger versus older patients are comparable, and suggest that patient age alone is not a relevant consideration for avelumab + axitinib treatment.

Data obtained from PMS have some limitations. First, source data from case report forms were not verified using medical records, per Good Post‐Marketing Study Practice ordinance. Second, methods of assessment in this PMS differed from those employed in the JAVELIN Renal 101 trial, and data were not reviewed by an independent data monitoring committee; thus, comparisons with JAVELIN Renal 101 data must be interpreted with caution. Third, the PMS was noncomparative and observational and did not collect information on patients who did not receive avelumab + axitinib; therefore, it is not possible to confirm that observations were due to drug exposure.

In conclusion, PMS data reported here demonstrate the safety and effectiveness of avelumab + axitinib across age subgroups of patients with aRCC in clinical practice in Japan, including older patients. The favorable benefit–risk profile was generally consistent with that observed in previous clinical studies and provides further support for the continued use of avelumab + axitinib as a standard of care in this setting.

## AUTHOR CONTRIBUTIONS

**Mototsugu Oya:** Conceptualization (equal); investigation (equal); supervision (equal). **Taito Ito:** Conceptualization (equal); methodology (equal); project administration (equal). **Masashi Sato:** Data curation (equal); formal analysis (equal); methodology (equal). **Makiko Morita:** Validation (equal). **Masahiro Kajita:** Conceptualization (equal); visualization (equal); writing – original draft (equal). **Norio Nonomura:** Conceptualization (equal); investigation (equal); supervision (equal).

## FUNDING INFORMATION

This study was sponsored by Merck Biopharma Co., Ltd., Tokyo, Japan, an affiliate of Merck KGaA, Darmstadt, Germany (CrossRef Funder ID: 10.13039/100009945), and was previously conducted under an alliance between the healthcare business of Merck KGaA, Darmstadt, Germany, and Pfizer.

## CONFLICT OF INTEREST STATEMENT

Mototsugu Oya has received honoraria from Bayer, Bristol Myers Squibb, Eisai, Merck Biopharma Co., Ltd., Tokyo, Japan, an affiliate of Merck KGaA, Darmstadt, Germany, Merck & Co., Kenilworth, NJ, Ono, Pfizer, and Takeda; has received manuscript fees from Pfizer; and has received scholarship endowments from Bayer, Eisai, Ono, and Takeda. Taito Ito, Masashi Sato, Makiko Morita, and Masahiro Kajita report employment with Merck Biopharma Co., Ltd., Tokyo, Japan, an affiliate of Merck KGaA, Darmstadt, Germany. Norio Nonomura has received royalties from Shionogi; has received honoraria from Astellas, AstraZeneca, Janssen, Merck Biopharma Co., Ltd., Tokyo, Japan, an affiliate of Merck KGaA, Darmstadt, Germany, and Takeda; has received research funds from Astellas, IQVIA, Parexel, and TOSOH; and has received scholarship endowments from Nippon Shinyaku, Ono, Pfizer, Taiho, Takeda, and Yakult.

## ETHICS STATEMENT

This PMS was conducted in accordance with Japanese regulations for Good Post‐Marketing Study Practice (GPSP). The protocol was reviewed by all participating institutions. The approval of the ethical committee/institutional review board and informed consent was obtained from patients based on requirements of each institution.

## Supporting information


**Table S1.** Dosing period, dosing frequency, and relative dose intensity.
**Table S2.** Summary of treatment discontinuation by age group.
**Table S3.** First infusion reaction by worst grade by age group.

## Data Availability

Any requests for data by qualified scientific and medical researchers for legitimate research purposes will be subject to the healthcare business of Merck KGaA, Darmstadt, Germany's Data Sharing Policy. All requests should be submitted in writing to the healthcare business of Merck KGaA, Darmstadt, Germany's data sharing portal (https://www.emdgroup.com/en/research/our‐approach‐to‐research‐and‐development/healthcare/clinical‐trials/commitment‐responsible‐data‐sharing.html). When the healthcare business of Merck KGaA, Darmstadt, Germany has a co‐research, co‐development, or co‐marketing or co‐promotion agreement, or when the product has been out‐licensed, the responsibility for disclosure might be dependent on the agreement between parties. Under these circumstances, the healthcare business of Merck KGaA, Darmstadt, Germany will endeavor to gain agreement to share data in response to requests.
