# Supplementary material for: Avelumab + axitinib treatment in older patients with advanced renal cell carcinoma in Japan: Subgroup analyses of post‐marketing surveillance data by age
Source: Cancer Med. 2025 Jan 21;14(2):e70186. doi: 10.1002/cam4.70186 (PMC11750686; doi:10.1002/cam4.70186)
Supplement: Supplementary file 1 — Table S1. Dosing period, dosing frequency, and relative dose intensity. Table S2. Summary of treatment discontinuation by age group. Table S3. First infusion reaction by worst grade by age group. [file CAM4-14-e70186-s001.docx]

**Table S1. Dosing period, dosing frequency, and relative dose intensity.**

|  | **≤64 years (n=100)** | | **65-74 years (n=130)** | | **≥75 years (n=98)** | |
| --- | --- | --- | --- | --- | --- | --- |
|  | **Avelumab**  **(n=100)** | **Axitinib**  **(n=100)** | **Avelumab**  **(n=130)** | **Axitinib**  **(n=130)** | **Avelumab**  **(n=98)** | **Axitinib**  **(n=97)** |
| **Total number of infusions received, median (range)** | 14.5  (1.0-26.0) | - | 15.0  (1.0-27.0) | - | 10.5  (1.0-26.0) | - |
| **Duration of treatment, median (range), months** | 8.2  (0.5-12.4) | 7.6  (0.1-12.6) | 8.8  (0.5-12.4) | 7.5  (0-12.8) | 5.5  (0.5-12.4) | 4.4  (0-12.7) |
| **Patients with dose reduction, n (%)** | 2  (2.0) | 50  (50.0) | 1  (0.8) | 75  (57.7) | 1  (1.0) | 52  (53.6) |
| **Patients with dose escalation, n (%)** | 0 | 16  (16.0) | 2  (1.5) | 22  (16.9) | 0 | 16  (16.5) |
| **Relative dose intensity, median (range), %** | 100.0  (64.4-101.0) | 88.3  (30.9-182.5) | 100.0  (20.0-100.0) | 76.4  (20.0-145.3) | 100.0  (80.0-100.0) | 70.9  (20.0-114.3) |

**Table S2. Summary of treatment discontinuation by age group.**

|  | **≤64 years (n=100)** | **65-74 years (n=130)** | **≥75 years (n=98)** |
| --- | --- | --- | --- |
| **Treatment continuations at data cutoff, n (%)** | 33 (33.0) | 50 (38.5) | 33 (33.7) |
| **Treatment discontinuation, n (%)**  Disease progression  ADR of safety specification  Surgery  Patient preference  Death  Transfer to another hospital  Other | 67 (67.0)  33 (33.0)  15 (15.0)  5 (5.0)  1 (1.0)  2 (2.0)  2 (2.0)  12 (12.0) | 80 (61.5)  25 (19.2)  19 (14.6)  8 (6.2)  6 (4.6)  7 (5.4)  2 (1.5)  13 (10.0) | 65 (66.3)  10 (10.2)  24 (24.5)  5 (5.1)  9 (9.2)  4 (4.1)  6 (6.1)  8 (8.2) |

Patients with >1 reason for discontinuation are counted in all relevant rows.

**ADR**, adverse drug reaction.

**Table S3. First infusion reaction by worst grade by age group.**

| **Dose, n (%)** | **≤64 years (n=100)** | | **65-74 years (n=130)** | | **≥75 years (n=98)** | |
| --- | --- | --- | --- | --- | --- | --- |
|  | **Grade 1-2** | **Grade ≥3** | **Grade 1-2** | **Grade ≥3** | **Grade 1-2** | **Grade ≥3** |
| First | 17 (17.0) | 0 | 17 (13.1) | 2 (1.5) | 19 (19.4) | 3 (3.1) |
| Second | 1 (1.0) | 0 | 0 | 1 (0.8) | 2 (2.0) | 0 |
| Third | 0 | 0 | 0 | 0 | 1 (1.0) | 0 |
| ≥Fourth | 1 (1.0) | 0 | 1 (0.8) | 0 | 0 | 0 |
| **Time of first infusion reaction onset** |  | |  | |  | |
| Immediately after the start of administration | 1 (1.0) | | 1 (0.8) | | 0 | |
| During administration | 5 (5.0) | | 12 (9.2) | | 8 (8.2) | |
| ≤1 hour after administration | 3 (3.0) | | 4 (3.1) | | 9 (9.2) | |
| >1 hour after administration | 10 (10.0) | | 4 (3.1) | | 8 (8.2) | |
| **Patients with >1 infusion reaction** | 0 | | 3 (2.3) | | 3 (3.1) | |
